# Identification of Transcriptional and Metabolic Programs Related to Mammalian Cell Size

**DOI:** 10.1016/j.cub.2014.01.071

**Published:** 2014-03-17

**Authors:** Teemu P. Miettinen, Heli K.J. Pessa, Matias J. Caldez, Tobias Fuhrer, M. Kasim Diril, Uwe Sauer, Philipp Kaldis, Mikael Björklund

**Affiliations:** 1 Division of Cell and Developmental Biology, College of Life Sciences, University of Dundee, Dundee DD1 5EH, UK; 2 Institute of Molecular and Cell Biology, Agency for Science, Technology and Research, 61 Biopolis Drive, Proteos #03–09, Singapore 138673, Singapore; 3 Department of Biochemistry, National University of Singapore, Singapore 117597, Singapore; 4 Institute of Molecular Systems Biology, Eidgenössische Technische Hochschule Zürich, Wolfgang-Pauli Strasse 16, 8093 Zürich, Switzerland

## Abstract

**Background:**

Regulation of cell size requires coordination of growth and proliferation. Conditional loss of cyclin-dependent kinase 1 in mice permits hepatocyte growth without cell division, allowing us to study cell size in vivo using transcriptomics and metabolomics.

**Results:**

Larger cells displayed increased expression of cytoskeletal genes but unexpectedly repressed expression of many genes involved in mitochondrial functions. This effect appears to be cell autonomous because cultured *Drosophila* cells induced to increase cell size displayed a similar gene-expression pattern. Larger hepatocytes also displayed a reduction in the expression of lipogenic transcription factors, especially sterol-regulatory element binding proteins. Inhibition of mitochondrial functions and lipid biosynthesis, which is dependent on mitochondrial metabolism, increased the cell size with reciprocal effects on cell proliferation in several cell lines.

**Conclusions:**

We uncover that large cell-size increase is accompanied by downregulation of mitochondrial gene expression, similar to that observed in diabetic individuals. Mitochondrial metabolism and lipid synthesis are used to couple cell size and cell proliferation. This regulatory mechanism may provide a possible mechanism for sensing metazoan cell size.

## Highlights

• Gene expression and metabolites levels relative to cell size are analyzed in liver
• Mitochondrial gene expression is repressed cell-autonomously in larger cells
• Cell size can be modulated by targeting mitochondria functions and lipid synthesis
• Lipids are negative regulators of cell size because they promote cell proliferation

## Teaser

Miettinen et al. use conditional knockout of Cdk1 in mouse liver to explore how cell size impacts transcription and metabolism. They find that large cells are not bioenergetically challenged and that mitochondrial function and lipid biosynthesis are changed in response to cell size, possibly due to the differences in the surface-area-to-volume ratio.

## Introduction

Cell size can be increased by impeding with cell-cycle progression, increasing the rate of biosynthesis, or both. In unicellular organisms, cell size and proliferation are mainly controlled by nutrient levels, whereas regulation through growth and mitogenic and survival signals is additionally important in metazoan cells [1]. Cell size increases with ploidy in many organisms, although the mechanism behind this is elusive [2, 3]. *Saccharomyces cerevisiae* has been the predominant model used to study cell size [2, 4]. Genes affecting cell size have been identified through loss-of-function studies in yeast [5, 6] and *Drosophila* [7, 8], as well as through gene-expression studies of yeast cell-cycle mutants and strains with variable ploidy [9–11]. However, in mammals, practically all insights are derived from cultured cells with a focus in understanding whether there is an active cell-size control [12–14]. Mechanisms that affect cell size in vivo have received less attention, apart from the role of mTOR.

Liver is a homogenous tissue mainly composed of hepatocytes. Liver regenerates to its normal size after partial hepatectomy ([PH]; removal of ∼70% of the liver) through cell growth and division of the remaining cells. Interestingly, mouse liver with a cyclin-dependent kinase 1 (Cdk1) liver-specific knockout (Cdk1^Flox/Flox^ Albumin-Cre, hereafter named Cdk1^Liv−/−^) can also regenerate. However, this occurs in the absence of cell divisions, resulting in enlarged hepatocytes [15]. Because Cdk1 is essential for cell-cycle progression, this model separates growth and proliferation effects, allowing us to analyze how mammalian cells respond to cell-size changes in vivo. We identify how gene-expression and metabolite levels correlate with cell size and discover that both mitochondrial metabolism and lipid biosynthesis are used to couple cell size and cell proliferation.

## Results

### Correlation of Gene Expression and Metabolite Levels with Cell Size In Vivo

**Figure 1:**
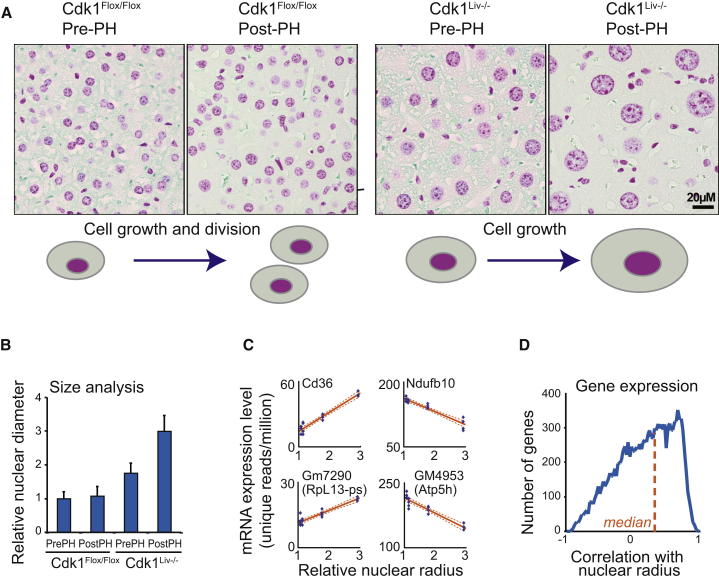
Correlation of Gene-Expression and Metabolite Levels with Cell Size in Mouse Liver (A) Representative Feulgen-stained histological sections of Cdk1^Flox/Flox^ and Cdk1^Liv−/−^ liver before and 96 hr after PH. The Cdk1^Liv−/−^ hepatocytes regenerate by growing in size because they are unable to divide, whereas the cell size in Cdk1^Flox/Flox^ liver is not significantly changed. All images were taken with the same magnification. Scale bar represents 20 μm. (B) Quantification of the nuclear sizes in liver samples. The data shown indicate mean ± SD of nuclear radius relative to control Cdk1^Flox/Flox^ before PH (n = 13–55 cells). (C) Analysis of gene expression by RNA-seq. Four genes displaying strong correlation with nuclear radius are shown as examples with correlation, and ±90% confidence intervals are shown with solid and dotted line, respectively. (D) A density plot of gene-expression correlations with nuclear radius for all genes. Median Pearson correlation (0.222) for all genes is indicated with the dotted line. See also Figure S1 and Tables S1 and S2.

Liver samples from control (Cdk1^Flox/Flox^) and Cdk1^Liv−/−^ animals, before and after partial hepatectomy, form a series of samples with different nuclear sizes (Figure 1A). Hepatocytes from Cdk1^Liv−/−^ mice after PH have 2–3 times larger radii than those from Cdk1^Flox/Flox^ mice ([15]; Figure 1B), with relatively uniform size increase because the variation is similar to controls (Figures 1A and 1B). We measured gene expression and relative metabolite levels in these four nearly isogenic sample types using nuclear radius as a proxy for cell size [2, 3]. We then correlated all gene expression and metabolite changes to cell size (Figures 1C and 1D; Figures S1A and S1B available online; Tables S1 and S2). Gene-expression data were validated by comparing samples before and after PH (Figure S1C) and by quantitative RT-PCR (Figures S1D and S1E). To our knowledge, there are no prior data regarding global gene expression and metabolic changes related to cell size from metazoan organisms in vivo.

The metabolomics data contained semiquantitative ion intensities, which potentially account for >2,200 metabolites based on accurate mass annotation and covering a large fraction of the metabolome (Figure S1F). We observed many changes related to hepatectomy (Figures S1B and S1G), including known changes in levels of glycogen, glucose, taurine, betaine, and creatine [16]. We could also identify changes related to Cdk1 deletion and cell size (Figure S1B). By plotting the correlation of the nuclear radius and change in metabolite and gene-expression levels between the largest and the smallest cells, we observed that the strongest correlations with cell-size change are usually not associated with the largest fold changes (Figure S1G; Tables S1 and S2).

At gene-expression level, the fatty acid transporter *Cd36* displayed almost perfect linear correlation (r = 0.968) with nuclear size (Figure 1C). We also identified genes with strong negative correlation (for example, *Ndufb10* [NADH dehydrogenase subunit, r = −0.947]) (Figure 1C), although these were less abundant (118 genes with correlation < −0.8 with nuclear size versus 302 genes with correlation > 0.8 with nuclear size). Such a coordinated global gene expression with cell size is consistent with yeast data [9, 10]. The distribution pattern of all gene and metabolite correlations with cell size is in Figures 1D and S1A. The observed gene-expression pattern could result from downregulation of a few highly expressed genes. However, the most abundant genes are on average only slightly downregulated, and the observed positive correlation is due to increased expression of many genes with low expression (Figure S1H). Because many of these are regulatory proteins, these changes might be necessary to support cell growth.

In contrast to yeast, in which G1 cyclins are repressed with increased cell size [9], the expression of many cell-cycle genes correlated positively with nuclear size. Cyclins D1–D3, E1, E2, A2, B1, and B2 displayed a positive correlation with cell size (r = 0.344–0.761; Table S1), suggesting that repression of cyclins is not universally required for cell-size increase.

### Mitochondrial and Cytoskeletal Genes Strongly Correlate with Cell Size

**Figure 2:**
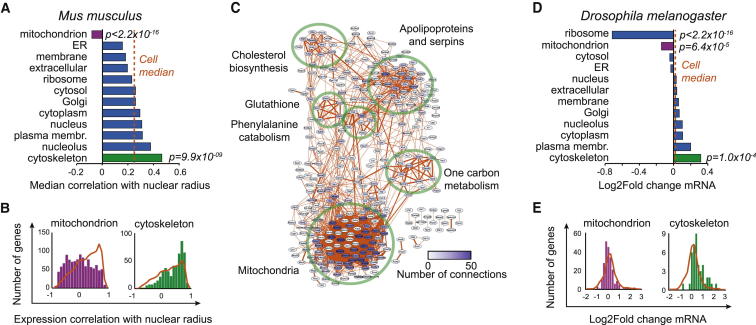
Correlation of Gene Expression with Cell Size for Different Subcellular Components Identifies Downregulation of Mitochondrial Genes (A) Mouse genes annotated to individual subcellular components using gene ontology (GO) analysis were identified, and median correlation with nuclear size was calculated. Dotted orange line indicates median cell correlation for all genes included in this analysis. We calculated p values using Kolmogorov-Smirnov test. (B) Expression correlations for genes annotated to mitochondria and cytoskeleton. Correlations were binned to obtain scaling profiles (bars) for each subcellular component. For comparison, the whole-cell profile (only genes with annotation in any of the subcellular component analyses, as opposed to all genes in Figure 1D, orange line) is overlaid on the bar chart. The number of genes in the whole-cell profile was normalized to the number of genes in individual subcellular components to simplify comparison. (C) Connectivity of genes correlating negatively (adjusted p value < 0.05) with cell size, as identified using the STRING database. Groups of functionally interacting genes are indicated with green circles and named. Note that one-carbon metabolism genes, such as adenosylhomocysteinase (*Ahcy*), are important for glutathione synthesis, indicating possible coregulation. (D) *Drosophila* genes annotated to individual subcellular components as for liver data. Dotted orange line indicates median of log_2_ fold change for all genes included in this analysis. (E) Histograms of mitochondrial and cytoskeletal gene expression compared to all genes (orange line) in *Drosophila* Kc167 cells. See also Figure S2 and Tables S3 and S4.

Rather than focusing on individual genes, we wanted to identify whether expression of genes related to various subcellular components is coordinated with cell size. Comparison of size correlation distributions for various subcellular structures based on gene ontology (GO) classifications revealed two structures that deviated from the whole-cell profile. These structures were cytoskeleton and mitochondria, correlating positively and negatively, respectively, with cell size (Figures 2A, 2B, and S2A). Because the cytoskeleton is required to mechanically support cells and is an integral part of various cellular transport mechanisms, the upregulation of cytoskeleton was not unexpected and has been observed in yeast [9]. Analysis of protein complexes indicated that the Wave2 and Arp2/3 complexes responsible for actin nucleation were among the most positively correlating complexes (Figure S2B). The negative correlation between mitochondrial gene expression and nuclear size was unexpected because mitochondrial deletion mutants in yeast display small cell size [5, 6], and mitochondrial content scales with cell size [17, 18]. The genes annotated in the inner membrane and matrix were the most negatively correlating gene sets within mitochondria (Figures S2C and S2D).

Next, we analyzed the connectivity of the genes correlating with cell size by using a protein-association network database. The positively correlating network contained DNA replication genes, ribosomal protein-coding genes, Rho GTPase-related genes, cytoskeleton and cell-adhesion-related genes, E2F-related, and Hippo pathway genes (Figure S2E), all of which are likely to be involved in growth. The negatively correlating network contained a large cluster of mitochondrial genes and smaller clusters containing cholesterol biosynthesis genes, apolipoprotein and serine protease inhibitors (serpin), and genes involved in glutathione, phenylalanine and tyrosine, and one-carbon metabolism (Figure 2C). These networks had 2.3 and 8.1 times more connections per gene, respectively, than similarly sized random networks, indicating functional interactions (Figure S2E). Many of these findings were corroborated by metabolomics data, which showed marked changes in glutathione, one-carbon, and DNA-replication-related metabolism (Table S3). Because one of the major functions of mitochondria is oxidative phosphorylation (OxPhos), we analyzed the expression of OxPhos genes. These displayed strong negative correlation with nuclear size (Figure S2F).

The identified gene-expression patterns could potentially be caused by Cdk1 deletion rather than by change in cell size. However, genes affected by Cdk1 deletion had very limited overlap with size-correlating genes. This overlap was only 4% (22 of 526) of positively correlating genes and 6% (36 of 569) of negatively correlating genes (Figure S2G), indicating that the observed effects are not Cdk1 dependent. Additionally, we used gene-expression data from cultured *Drosophila* Kc167 cells. Knockdown of *Pop2* deadenylase, which causes degradation of mRNA polyA tails, increases cell size ∼20% without major effects on cell cycle (Figures S2H and S2I). The CCR4-NOT complex, which contains a *Pop2* ortholog, has one of the strongest cell-size effects in yeast [6]. Analysis of *Drosophila* RNA-expression data (Table S4) indicated that mitochondrial genes were significantly downregulated and that cytoskeletal genes were upregulated (Figures 2D and 2E). The exception to liver data was that ribosomal gene expression was repressed, and this may be a feedback mechanism related to stabilization of mRNAs. The similarity of gene-expression signatures in mouse and *Drosophila* cells implies that these gene-expression changes are cell-autonomous effects related to cell size.

### Aerobic Glycolysis Fuels Cell Size Increases

**Figure 3:**
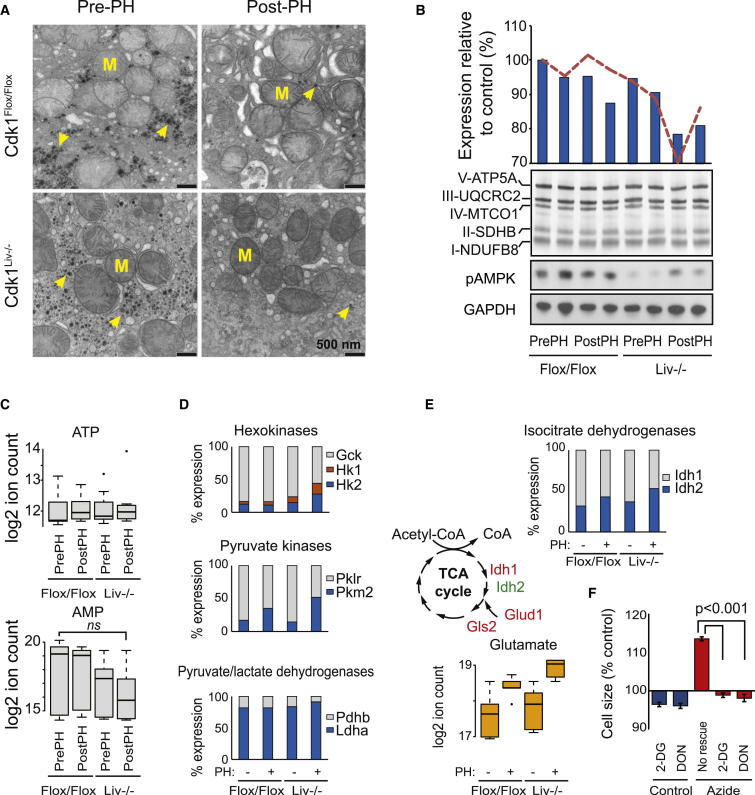
Glycolysis Increases with Cell Size (A) Representative electron microscopy images of Cdk1^Flox/Flox^ and Cdk1^Liv−/−^ liver before and after hepatectomy. Arrows and “M” indicate glycogen and mitochondria, respectively. All scale bars represent 500 nm. For quantification, see Figure S3A. (B) mRNA expression (red line) and protein levels (blue bars) of selected OxPhos proteins. Western blot shows the measured OxPhos complex components, phospho-Thr172-AMPK (pAMPK) levels, and GAPDH (loading control). (C) Relative ATP and AMP levels in liver samples, as measured by mass spectrometry. Statistical significance was measured by ANOVA. (D) Proportional expression of key glycolytic genes based on liver RNA-seq data. (E) Glutamate metabolite levels (orange) and Idh expression levels (blue and gray) correlate with cell size. (F) Inhibition of glycolysis and glutaminolysis by 2-DG and DON rescue U2OS cell size increase by 1 mM sodium azide (p < 0.001 in both; t test, mean ± SD, n = 3). See also Figure S3.

The negative correlation of mitochondrial gene expression with cell size suggested changes in energy metabolism. We did not observe significant changes in mitochondrial number, size, or number of cristae, although the mitochondria in larger cells tended to be smaller and slightly more abundant, with increased electron density (Figures 3A and S3A). Further analysis of the gene-expression levels of mitochondrial DNA-replication machinery (Figure S3B) and mitochondrial DNA amount relative to genomic DNA (Figure S3C) did not indicate depletion of mitochondria.

Despite negative correlation, the absolute reduction of mRNA and protein expression of OxPhos complexes in Cdk1^Liv−/−^ post-PH samples compared to Cdk1^Flox/Flox^ pre-PH samples was only ∼20% (Figure 3B), which could explain the phenotypic difference of yeast deletion mutants [5, 6]. Metabolomics data indicated no changes in ATP levels but a reduction in AMP levels, which correlated with cell size (Figure 3C). Consistently, analysis of the cellular energy sensor AMP-activated kinase (AMPK) indicated downregulation of AMPK activity in Cdk1^Liv−/−^ cells (Figure 3B). Thus, ATP levels are unlikely to be limiting in larger cells, and the lack of AMPK activation could provide permissive conditions for the cell-size increase. We conclude that the observed mitochondrial gene-expression correlation is due to moderate transcriptional downregulation.

Compensatory increases in glycolysis could maintain ATP levels, and we indeed observed upregulation of genes related to three key regulatory steps (Figure 3D). Hexokinase expression correlated well with cell size, whereas pyruvate kinase *Pkm2* displayed a mixed hepatectomy and cell-size effect. Additionally, lactate dehydrogenase (*Ldha*) correlated positively and pyruvate dehydrogenase (*Pdha*) correlated negatively with cell size (Figure 3D). Analysis of the metabolite levels indicated that, whereas changes in glucose levels in control and Cdk1^Liv−/−^ animals were roughly similar, changes caused by PH in pyruvate levels at the end of the glycolytic pathway were different (Figure S3D). For a summary of all glycolysis data, see Figures S3D and S3E.

We did not observe significant changes in tricarboxylic acid (TCA) cycle metabolites (Figure S3D), although we observed changes in isocitrate dehydrogenases 1 and 2 (*Idh1* and *Idh2*) as well as in mitochondrial glutaminase 2 (*Gls2*) and glutamate dehydrogenase 1 (*Glud1*), with concomitant increase in glutamate levels (Figure 3E). The glycolytic inhibitor 2-deoxyglucose (2-DG) and the glutamine antagonist 6-Diazo-5-oxo-L-norleucine (DON) abolished the cell-size increase caused by respiratory inhibitor sodium azide in human osteosarcoma cell line U2OS (Figure 3F). Altogether, these data suggest that glycolysis and glutaminolysis are required to fuel cell growth caused by mitochondrial inhibition (by sodium azide) in vitro and possibly in vivo.

Interestingly, although metabolic changes in early glycolysis displayed a clear hepatectomy effect, Cdk1^Liv−/−^ genotype and cell size had more effect on metabolite levels at later stages of glycolysis. For example, we observed that Cdk1^Liv−/−^ suppressed the increased pyruvate levels caused by hepatectomy in control animals (Figures S3D and S3E). Furthermore, hepatectomized Cdk1^Liv−/−^ knockout mice displayed increased metabolite levels related to serine and glycerol synthesis. These metabolic changes and the positive correlation of pyruvate kinase *Pkm2* expression with cell size are consistent with tumor-like metabolic phenotype [19]. Overall, the observed cell-size-related metabolic and gene-expression changes are conceptually similar to the Warburg effect, in which mitochondrial activity is reduced relative to glycolysis.

### Mitochondria Regulate the Balance between Cell Size and Cell Proliferation

**Figure 4:**
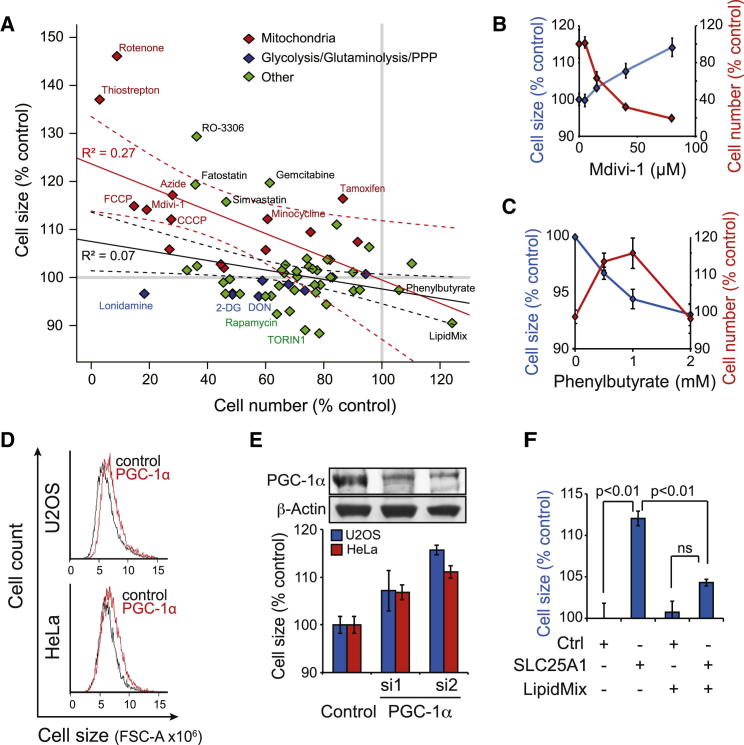
Inhibition of Mitochondrial Functions Increases Cell Size in Cultured Cells (A) Changes in cell size and cell number in U2OS cells by small molecules. Compounds with known effects on mitochondria are displayed in red. Glycolysis, glutaminolysis, and PPP compounds are displayed in blue, and others are displayed in green. Red and black solid lines display linear regression for mitochondria targeting and for all other compounds, respectively, with 90% confidence intervals shown as dotted line. See Table S5 for all compounds and concentrations used. (B) U2OS cell number (red line) and cell size (blue line) were analyzed as a function of Mdivi-1 concentration (n = 3, 48 hr). (C) HeLa cell number (red line) and cell size (blue line) as a function of phenylbutyrate concentration in delipidated FBS (n = 3, 48 hr). (D) Representative cell-size profiles for PGC-1α knockdown in U2OS and HeLa cells. (E) Quantification of cell-size changes by two PGC-1α targeting siRNAs (25 nM) compared to control RNAi-treated cells (n = 3, 48 hr), with a western blot showing the knockdown efficiency in U2OS cells. All treatments except siRNA1 in U2OS cells had p value < 0.01 (t test). (F) Rescue of SLC25A1 RNAi (15 nM) by LipidMix (50 μl/ml) (n = 3, 48 hr). Data shown indicate mean ± SD with t test (ns, not significant). See also Figure S4 and Table S5.

To investigate this putative functional link between mitochondria and cell size, we screened a set of small molecules, including compounds that target mitochondria and glycolysis, glutaminolysis, and the pentose phosphate pathway (PPP). Mitochondria-targeting inhibitors frequently increased cell size and reduced cell numbers, with a modest inverse correlation (R^2^ = 0.27) (Figure 4A). The mitochondria-targeting compounds included uncoupling agents (FCCP and CCCP), ionophore (valinomycin), mitochondrial division inhibitor (Mdivi-1), translation inhibitors (minocycline and thiostrepton), and drugs with mitochondrial off-targets (tamoxifen).

The reciprocal effects on cell size and proliferation are illustrated with Mdivi-1, which targets the dynamin-related protein 1 (Drp1), and sodium azide, an inhibitor of OxPhos complex IV (Figures 4B, S4A, and S4B). Increases in cell size were similarly detected by electrical current exclusion method and by measurement of protein amount per cell, arguing against osmotic effects (Figures S4C and S4D). In contrast to mitochondrial inhibitors, phenylbutyrate, which enhances the metabolic flux from glycolysis to mitochondria, caused increased proliferation and decreased cell size, although it slowed down proliferation at high concentrations (Figure 4C). Most nonmitochondria-targeted chemicals had little effect on cell size, although they reduced cell number and consequently displayed no correlation with cell size (R^2^ = 0.07) (Figure 4A). These data, together with our RNAi screen in *Drosophila* [7] and recent yeast data [20], indicate that cell size is, in most cases, not connected to effects in cell proliferation (cell cycle) as commonly believed.

Genetic means of targeting mitochondrial functions also increased cell size. U2OS^rho0^ cells, which do not contain mitochondrial DNA and thus are defective in many mitochondrial functions, were larger than wild-type cells (Figure S4E). Cell size was also increased by RNAi of the transcriptional coactivator PGC-1α (Figures 4D and 4E), which has been implicated as an integrator of metabolism and mitochondrial gene expression by regulating OxPhos, TCA cycle, and lipid synthesis genes.

Because two OxPhos complex inhibitors, antimycin A and oligomycin, did not increase cell size (Table S5 and Figure S4F), we examined what other mitochondrial functions could explain the cell-size phenotype. Mitochondrial metabolism is closely linked to oxidative phosphorylation and proliferation [19, 21]. A key function of mitochondria is to provide acetyl-coenzyme A (CoA) for histone acetylation as well as for mevalonate and cholesterol and fatty acid synthesis (Figure S4G). Mitochondrial acetyl-CoA is exported to the cytoplasm as citrate. Although our metabolomic data cannot distinguish subcellular pools of metabolites, all enzymes involved in the citrate and acetyl-CoA transport process correlated negatively with cell size in our gene-expression data (Figure S4G). Additionally, RNAi of the mitochondrial citrate transporter SLC25A1 increased the size of U2OS and HeLa cells (Figure S4H).

In yeast, acetylation of histones binding to growth gene loci is important for promoting transcription and inducing proliferation [22]. We thus tested whether the cell-size effects in our models are linked to histone acetylation. Histone acetylation was reduced in larger Cdk1^Liv−/−^ cells in vivo (Figure S4I), as well as in U2OS cells treated with rotenone, an OxPhos complex I inhibitor and a potent cell-size inducer (Figure S4J). However, although all of the histone acetyltransferase inhibitors that were tested reduced cell proliferation, none of these inhibitors increased cell size (Figure S4K). Thus, histone acetylation levels are important for cell proliferation but do not explain cell size increases.

Because mitochondrially derived acetyl-CoA is also used for lipid biosynthesis, we attempted to rescue citrate transporter SLC25A1 RNAi by supplementing U2OS cells with a commercially available lipid mixture (LipidMix). This almost completely rescued the cell-size increase caused by SLC25A1 RNAi (Figure 4F). Interestingly, the effect of Mdivi-1 was also rescued by addition of LipidMix (Figure S4L).

### Repression of Lipid Biosynthesis Increases Cell Size

**Figure 5:**
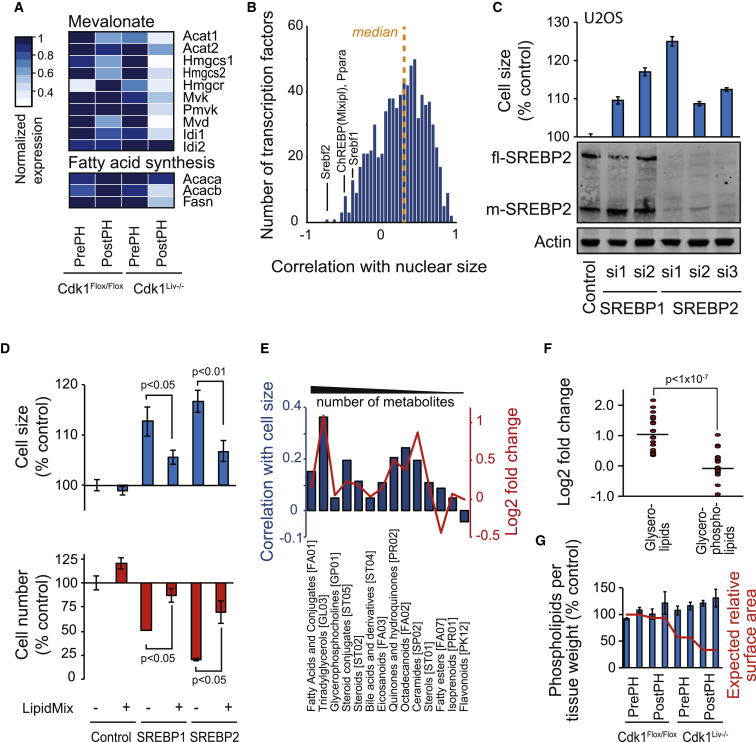
SREBP-Mediated Lipid Biosynthesis Is Involved in Modulation of Cell Size (A) Relative expression of genes in the mevalonate and cholesterol synthesis pathway and fatty acid synthesis pathway decreases with cell size in mouse liver. The expression values were normalized to the highest expression for each gene. (B) Histogram of individual transcription factor expression correlation with cell size in mouse liver. Median correlation of all transcription factors (r = 0.275) is indicated with the dotted line. (C) Quantification of U2OS cell-size changes by targeting SREBP1 and SREBP2 with nonoverlapping siRNAs (25 nM, n = 3, 60 hr). Knockdown of SREBP2 was validated by western blotting. β-actin was used as loading control. Compared to control, p value < 0.001 with all SREBP siRNAs (t test). (D) Rescue of cell size by SREBP RNAi using LipidMix in U2OS cells. Significance was analyzed by t test (n = 3, 55 hr). (E) Correlations (blue bars) and log_2_ fold changes (red line) for all lipid classes containing more than four metabolites, as classified in LIPID MAPS (http://www.lipidmaps.org). (F) Log_2_ fold changes between smallest and largest liver cells for individual glycerolipids and glycerophospholipids based on the metabolomics measurement. Horizontal line indicates mean (t test). (G) Measurement of total phospholipids using a colorimetric assay from liver extracts. Phospholipids were normalized to tissue weight. Expected cell-surface area relative to volume is in red. The differences in phospholipid levels are significant (p < 0.01, ANOVA). Data shown in (C), (D), and (G) indicate mean ± SD (n = 3). See also Figure S5.

We considered whether coupling of lipid synthesis and cell proliferation could explain our observations of mitochondria and cell size. Gene expression related to de novo lipid biosynthesis negatively correlated with cell size (Figure 5A). Analysis of individual transcription factors identified the sterol-regulatory element binding transcription factor 2 (SREBF2/SREBP2) as the most negatively correlating (Figure 5B). Analysis of transcription factor families identified E2F, ARID, and ETS factors correlating positively and STATs and PPARs correlating negatively with cell size (Figure S5A). Interestingly, 17 out of 55 transcription factors with a negative size correlation of <−0.3 clustered based on network analysis (Figure S5B), and these are involved in regulation of lipid metabolism either directly (SREBPs, PPAR-α, retinoic acid receptors, LXR/Nr1h2, ChREBP/Mlxipl, and HNF4A) or via inflammatory responses (STATs and IRFs). The coordinated downregulation of this network demonstrates the well-known crosstalk between metabolic and inflammatory signals [23], which is clinically important in diabetes, obesity, and atherosclerosis.

SREBP1 preferentially activates fatty acid metabolism, whereas SREBP2 activates cholesterol metabolism [24], and the activities of SREBP1 and SREBP2 are regulated by phosphatidylcholine and cholesterol, respectively [24, 25]. Expression of genes involved in SREBP maturation was also negatively correlated (Figure S5C). Because cholesterol synthesis and one-carbon metabolism are SREBP targets [25], this likely explains their negative correlation of gene-expression and metabolite levels with cell size (Figure 2C and Table S3B).

RNAi of both SREBP1 and SREBP2 increased cell size in U2OS and hTERT-RPE cells (Figures 5C and S5D). The effect was dose dependent (Figure S5E), and silencing of SREBP1 and SREBP2 increased cell size more than either treatment alone (Figure S5F). It has been reported that SREBP RNAi decreases the size of RPE cells [26], but this observation may be due to the combined effects of AKT and hydroxytamoxifen (note that tamoxifen potently increases cell size) or to more complete knockdown because SREBP knockout mice are lethal. Importantly, the SREBP1 and SREBP2 knockdown-induced cell-size increase in our experiments could be seen with multiple small interfering RNAs (siRNAs) and could be rescued with lipid mixture, making it unlikely that this is an off-target effect (Figures 5C and 5D).

Of all lipid classes, triacylglycerides displayed the best correlation with cell size (Figure 5E and Table S3C), and this accumulation of lipids may explain the downregulation of the lipogenic transcription factors [24, 25]. Although accumulation of hepatic lipids may lead to fatty liver, Cdk1^Liv−/−^ mice do not have fatty liver disease based on PPARγ expression (Figures S5G and S5I).

Increased cell size should result in a decrease of the relative surface area compared to volume. Metabolomics data indicated that the total levels of the detected phospholipids (reflecting membrane synthesis) were not changed in larger cells, whereas storage lipids (triglycerides) were clearly increased (Figures 5E and 5F). Direct measurement of total phospholipids in liver samples displayed a minor increase (Figure 5G). We also reanalyzed a yeast lipidomics experiment [27] that used haploid and diploid cells. These data revealed that two of the three most abundant phospholipids are increased in larger cells. It appears that total phospholipids, which are present in both plasma membrane and internal membranes, increase with cell size, and the change in phospholipid levels does not match the change in cell-surface area (Figure 5G).

**Figure 6:**
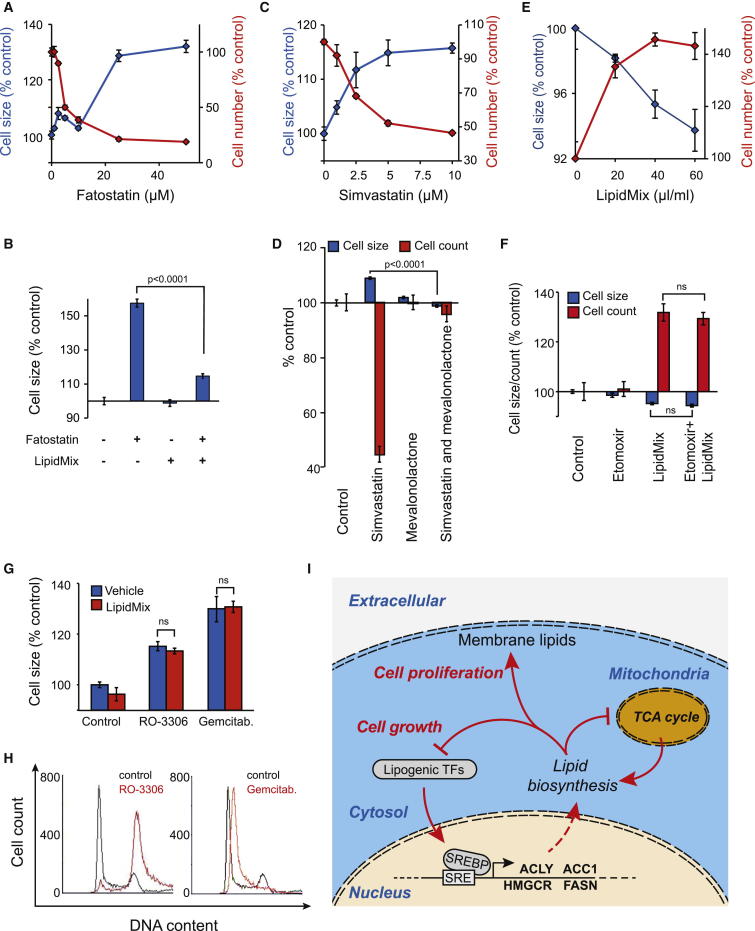
Lipids Modulate Cell Size and Proliferation Ratio (A) Cell number (red line) and cell size (blue line) were measured after 48 hr (n = 3). (B) Fatostatin (25 μM) effects on U2OS cell size were rescued by 50 μl/ml LipidMix (n = 3, 64 hr, t test). (C) U2OS cell number (red line) and cell size (blue line) were analyzed as a function of simvastatin concentration (n = 3, 48 hr). (D) Simvastatin (7.5 μM) effects on U2OS cell size and cell proliferation were rescued by 5 mM mevalonolactone (n = 3, 60 hr, t test). (E) Dose dependence of increased HeLa cell proliferation by LipidMix in delipidated FBS-containing medium (n = 3, 48 hr). (F) Effect of etomoxir (50 μM) on LipidMix induced HeLa cell proliferation in 10% lipid-free FBS (n = 3, 60 hr, t test). (G) Effect of LipidMix (50 μl/ml) on cell size in U2OS cells arrested with 7.5 μM RO-3306 or 1 μM Gemcitabine (n = 3, 34 hr). Data shown in (A)–(G) indicate mean ± SD. (H) Cell-cycle arrests in G2/M and early S phase by RO-3306 and Gemcitabine, respectively, were verified by DNA staining. (I) When cells proliferate, high mitochondrial metabolic activity and lipogenic transcription-factor levels are maintained. When cell size increases, the relative need for plasma membrane lipids decreases. Intracellularly accumulating lipids repress the activity of lipogenic transcription factors and, consequently, lipid synthesis-related gene expression. Downregulated lipid biosynthesis, in turn, reduces the need for mitochondrial metabolism. Similarly, if mitochondria are inhibited, proliferation is reduced without directly inhibiting cell growth. SREBP is shown as an example; SRE is sterol-regulatory element DNA motif. See also Figure S6.

To further investigate the relationship between lipids and cell size, we inhibited SREBP processing using fatostatin. Fatostatin increased cell size with a reduction in cell number in multiple cell lines (Figures 6A and S6A). This effect was almost completely rescued by lipid addition (Figure 6B). Similar effects were obtained by inhibition of cholesterol synthesis by simvastatin, which inhibits HMG-CoA reductase (Figure 6C). Specific rescue of simvastatin was obtained by mevalonolactone, the end product of the reaction (Figures 6D and S6B). Opposite effects on cell proliferation and cell size could be seen by supplying HeLa cells grown in lipid-depleted fetal bovine serum (FBS) with LipidMix. This treatment dose-dependently increased cell number and reduced cell size (Figure 6E). This was also observed to a lesser extent with normal FBS, although higher concentrations of lipids caused lipotoxicity (Figure S6C). The lipids were not used as an energy source because inhibition of beta-oxidation with etomoxir could not rescue this effect (Figure 6F). Hence, the lipids are either used as building blocks for membrane or for signaling to the cell-proliferation machinery.

To further demonstrate that lipids regulate the balance between cell size and cell proliferation, we increased cell size by blocking cell-cycle progression using Cdk inhibitor RO-3306 and DNA synthesis inhibitor gemcitabine (Figures 6G and 6H). Although these treatments significantly increased cell size, cell size could not be rescued by LipidMix. Thus, cell-cycle arrest and mitochondria-mediated increases in cell size are distinct, and LipidMix supplementation does not inhibit cell-size increase but acts by stimulating cell proliferation.

In diabetic and/or obese patients, excess lipids are associated with both the decline in mitochondrial functions and the decline in mitochondrial gene expression [28–30]. We observed that inhibition of SREBP function with fatostatin and SREBP RNAi resulted in increased mitochondrial membrane potential (Figures S6D and S6E), indicative of the well-known feedback mechanism between lipids and mitochondrial function. Altogether, our data validate a role for mitochondria and lipids in regulating the balance between cell size and cell proliferation.

## Discussion

We have investigated how mouse liver cells respond to increased cell size caused by Cdk1 inactivation and hepatectomy. Whereas cytoskeletal gene expression positively correlates with cell size, unexpectedly, the expression of mitochondrial and de novo lipid biosynthesis genes inversely correlates with cell size. Inhibition of mitochondrial functions and lipid synthesis increases cell size in culture, suggesting causality. Although decline in nutrient transport efficiency and increase in time required for diffusion-limited processes could potentially limit cell size, Cdk1^Liv−/−^ cells grow without signs of energy deprivation. The liver and *Drosophila* models indicate that the observed mitochondrial link is not Cdk1 dependent or cell-cycle dependent and that it is a cell-autonomous response related to cell size.

A fundamental unresolved issue in cell biology is the coupling of cell size and cell proliferation. We demonstrate that the balance between cell size and cell proliferation can be changed by targeting mitochondria and/or lipid biosynthesis, providing one possible mechanism for this coupling. Many mitochondrial and lipid metabolism genes are downregulated in proliferating hepatocytes in vivo [31]. However, because most proliferating cells grow in size before cell division, the complete lack of proliferation in our model allows separation of growth effects. Neutral lipids, such as triglycerides, substantially increase after PH [32], and growth immediately after hepatectomy occurs by cellular hypertrophy before initiation of the cell division [33]. This physiological coincidence of growth with lipid accumulation before hepatocyte proliferation is consistent with our data.

The mitochondrial and glycolytic changes observed in our model bear similarities to the Warburg effect. Our data suggest that the Warburg effect is primarily driving cell growth and not proliferation, as is often thought. Our findings are supported by a recent study in which the Warburg effect is not needed for T cell proliferation [34]. Furthermore, increased aerobic glycolysis decouples cell proliferation and biomass production in yeast [35], and PGC-1α expression correlates with proliferation of melanoma cells [36]. It is possible that the change in glycolysis and mitochondrial activity related to cell size may be more related to optimization of metabolic-precursor production than energy production.

At first glance, our results conflict with mTOR effects. mTOR activity increases cell size and activates lipid biosynthesis, which increases proliferation and, together with increased protein biosynthesis, results in increased biomass. In our experiments, cell size is increased at the cost of reduced proliferation with no increase in biomass production. We also show that the opposite is true by supplying cells with lipids, which reduces cell size and increases proliferation. Our results are consistent with physical laws in which increased cell size is expected to result in relative reduction of plasma membrane production as volume grows faster than surface area (r^3^ versus r^2^, where r is radius) and generates a scaling problem for a growing cell. The physical scaling problem assumes that lipids are used for plasma membrane production and does not take internal membranes into account. Our analysis suggests that there is an increase in internal membranes, in membrane composition, or in free phospholipids because total phospholipid levels are greater in larger cells. On the other hand, cholesterol is highly plasma membrane enriched, and the most affected transcription factor was *SREBPF2*, which is responsible for cholesterol biosynthesis. To understand the role of lipids in the scaling problem, we will need to measure quantities and membrane selectivity of individual lipid species in detail.

In summary, increased cell size results in the relative reduction of mitochondrial metabolism and lipid biosynthesis in mouse liver (Figure 6I). When proliferation is reduced, the need for plasma membrane components is reduced, and excess lipids, which are not incorporated into the plasma membrane, accumulate and inhibit lipogenic transcription factors. This reduces lipid biosynthesis and, consequently, the need for mitochondrial metabolism. This negative feedback thus matches the cell-size and cell-proliferation rate and may provide a solution for the scaling problem. Thus, lipids, which are not incorporated into membranes, can potentially be part of a cell-size-sensing mechanism.

## Experimental Procedures

The complete details of the experimental procedures are provided in the Supplemental Experimental Procedures.

Cdk1 conditional knockout mice have been described previously [15]. All the procedures performed were in accordance with institutional guidelines at the Institute of Molecular and Cell Biology, A^∗^STAR, Singapore. Livers were collected before and 96 hr after partial (70%) hepatectomy. Nuclear size was calculated from histological sections and normalized to Cdk1^Flox/Flox^ mice before hepatectomy. Pearson correlation coefficients were calculated using all samples for each gene and metabolite.

Liver samples were analyzed by RNA sequencing (RNA-seq) and by mass spectrometry for metabolomics. Cell-size and cell-number measurements were conducted using flow cytometry. Small molecules were from Sigma-Aldrich, Tocris, Santa-Cruz, or Calbiochem. RNAi was performed by transfecting siRNA with HiPerfect (QIAGEN). The siRNA and oligonucleotide sequences are in Table S6. Antibodies were detected using infrared-dye conjugated secondary antibodies and LICOR Odyssey detection system. For electron microscopy, liver pieces were fixed and exposed to osmium tetroxide and then embedded in Spurr’s resin.

## Accession Numbers

The ArrayExpress Archive accession number for the liver gene-expression data in this paper is E-MTAB-1297.

## Supplemental Information

Document S1. Supplemental Experimental Procedures and Figures S1–S6
