# Supplementary material for: Identification of Transcriptional and Metabolic Programs Related to Mammalian Cell Size
Source: Curr Biol. 2014 Mar 17;24(6):598–608. doi: 10.1016/j.cub.2014.01.071 (PMC3991852; doi:10.1016/j.cub.2014.01.071)
Supplement: Document S1. Supplemental Experimental Procedures and Figures S1–S6 [file mmc1.pdf]

Current Biology, Volume *24*

Supplemental Information

Identification of Transcriptional

and Metabolic Programs

Related to Mammalian Cell Size

Teemu P. Miettinen, Heli K.J. Pessa, Matias J. Caldez, Tobias Fuhrer, M. Kasim Diril, Uwe Sauer, Philipp Kaldis, and Mikael Björklund

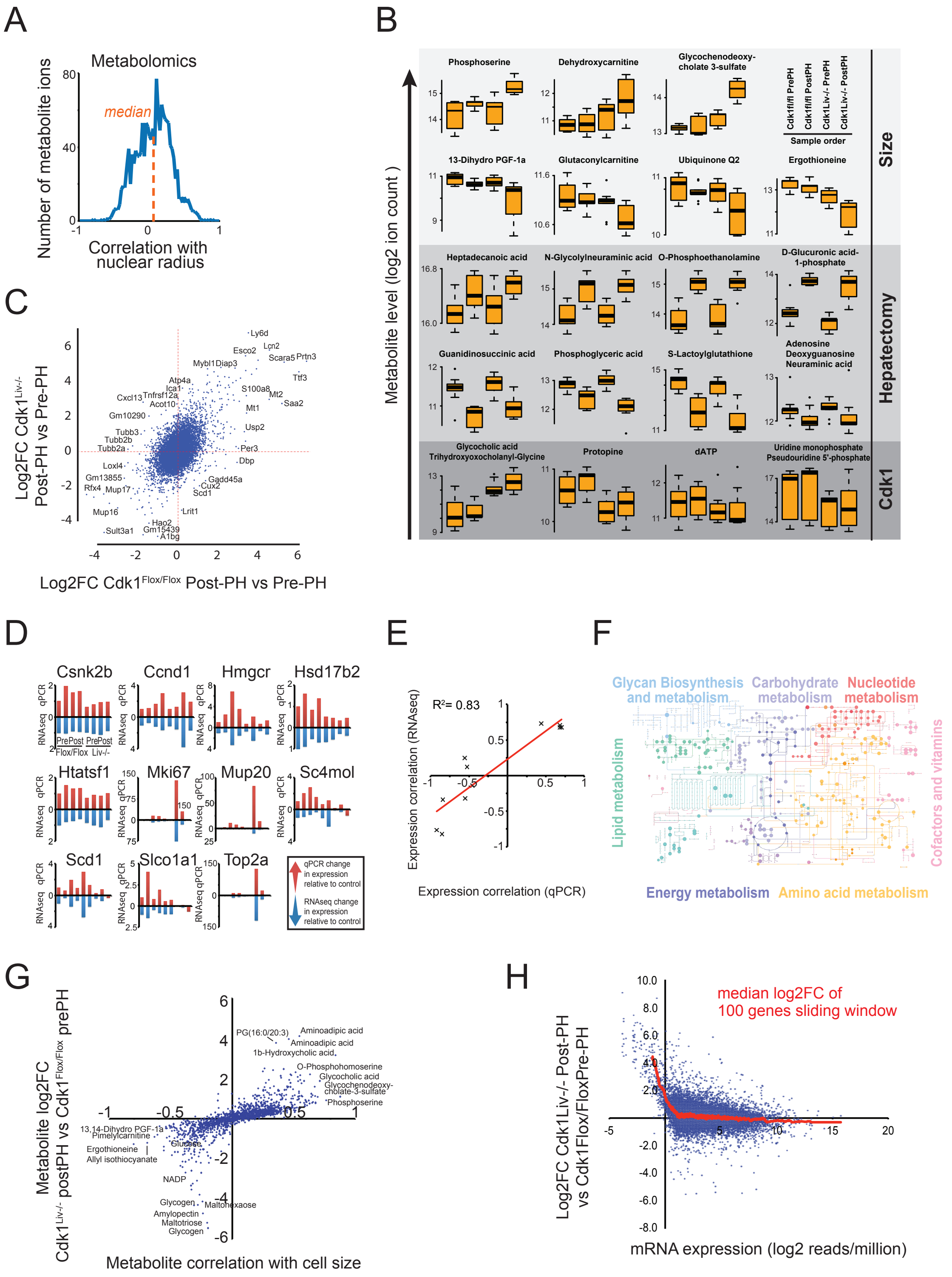

Figure S1, related to figure 1. Transcriptomics and metabolomics analysis of cell size *in vivo*. (A) Analysis of metabolite levels by mass spectrometry. A density plot of all metabolite level correlations with nuclear radius is shown. Median Pearson correlation (0.066) is

indicated with a dotted line. (B) Examples of metabolites displaying cell size, hepatectomy or Cdk1 effects. Note that we identified also metabolites such as ergothioneine, trehalose and protopine which are not produced by mammalian metabolism, but maybe derived from food or microbial metabolism. (C) Scatter plot of gene expression changes in response to hepatectomy in Cdk1$^{Flox/Flox}$ (x-axis) and Cdk1$^{Liv-/-}$ livers (y-axis). Mean values of the replicate samples were used to calculate fold-changes. As expected, we observed upregulation of Scara5, serum amyloid A and metallothionein genes in response to partial hepatectomy. (D) Validation of RNAseq data with quantitative RT-PCR analysis of a selection of genes. Mean expression levels relative to Cdk1$^{Flox/Flox}$ control animal before partial hepatectomy as measured by quantitative PCR (red bars) or RNAseq (blue bars). qPCR data shown is mean expression of three technical replicates for each liver sample. RNAseq data is calculated from the mean expression of technical replicates and plotted as negative values for clarity. The gene names are indicated above the individual histograms. Sample identities are shown in the Csnk2b graph only but are the same in all graphs. Note that in a few cases, qPCR shows reduced expression compared to RNAseq, e.g., the last bar in Sc4mol. (E) Correlation plot for Pearson correlation coefficients with nuclear radius for the genes shown in (D), as analyzed by qPCR (x-axis) and RNAseq (y-axis). The correlation between the two gene expression methods is shown (R^2 = 0.83). (F) Annotation coverage of the metabolomics data using 3 mD mass tolerance overlaid on the KEGG human metabolome map (hsa01100). The map is colored by pathways. Dot size reflects log10 average intensity of metabolite levels over all samples. (G) Scatter plot of metabolite ion correlations with nuclear/cell size and log2 fold changes between smallest and largest cells. Data is derived from both aqueous and organic extractions of the metabolomics data. (H) Comparison of mRNA expression levels (in Cdk1$^{flox/flox}$ livers before hepatectomy) and fold changes between smallest and largest cells. Red line indicates median log2 fold change of 100 genes

sliding window. Our data suggests that genes whose expression is low are most sensitive to cell size changes.

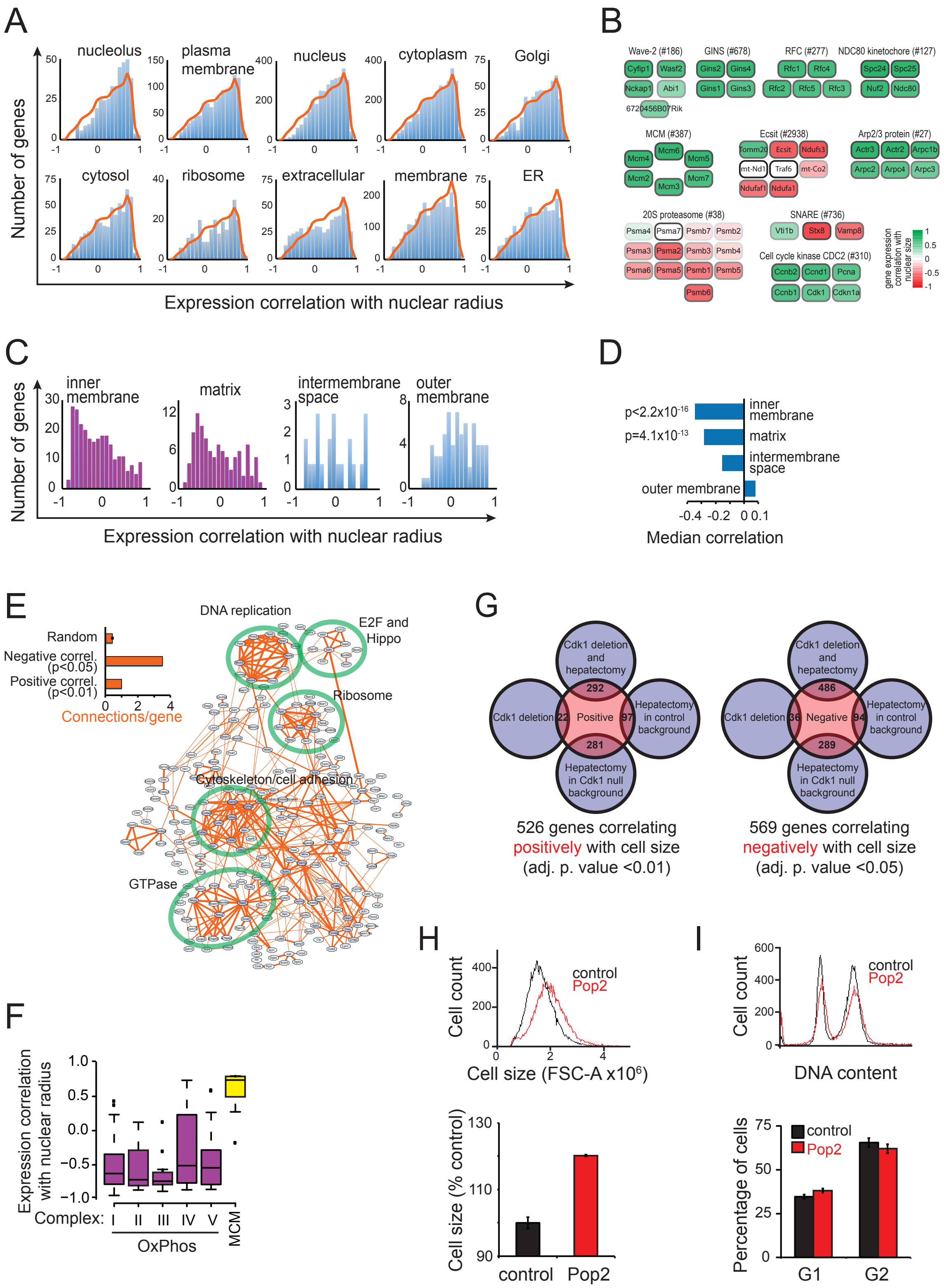

Figure S2, related to figure 2. Additional analysis of cell size gene expression programs. (A) Mouse liver mRNA expression correlations for genes annotated to individual

subcellular components were binned to obtain scaling profiles (bars) for each subcellular component. For comparison, the whole cell profile (all genes with annotation in any of the subcellular component, orange line) is overlaid on the bar chart. The number of genes in the whole cell profile was normalized to the number of genes in individual subcellular component to simplify comparison. Interestingly, plasma membrane annotated genes are not coordinately downregulated. (B) Examples of protein complexes, which gene expression positively and negatively correlates with cell size. The CORUM complex database numbers for each complex are indicated (mips.helmholtz-muenchen.de/genre/proj/corum). Note that we detect expression of Cdk1 mRNA as the knockout is a result of Cdk1 exon 3 deletion only in hepatocytes and the signal may stem from other cell types in the liver. (C) Analysis of gene expression profiles for mitochondrial substructures using GO analysis. (D) Summary of the mitochondrial gene expression changes. p values were calculated using Kolmogorov-Smirnov test. (E) Connectivity of genes correlating positively (adj.p.value <0.01) with cell size as identified using the STRING database. Groups of functionally interacting genes are indicated with green circles and named. Number of connections in positively and negatively correlating gene sets (Fig. 2C) as well as in similar sized random networks (mean and SD of five random networks) is show in the inset. Note that the number of connections in the gene set correlating negatively with cell size is more than three times higher than that of the positively correlating gene set. (F) Correlation of individual OxPhos complexes (I-V) and the minichromosome maintenance complex (MCM) with nuclear radius. Boxplot indicates median correlation. Outliers are shown with black dots. (G) Venn diagrams depicting the overlap between differentially expressed genes in pairwise sample and size correlating genes. Our data indicates a poor overlap between identified cell size genes and genes responding to Cdk1 deletion or partial hepatectomy. (H) Cell size histogram and relative cell change in dsRED control and Pop2 RNAi treated Kc167 cells. Cells were treated by RNAi for 4 days.

(I) Percentage of cells in G1 and G2 in dsRED and Pop2 RNAi treated Kc167 cells used for *Drosophila* RNAseq analysis. G1 and G2 populations were analysed from DNA content histograms. Data shown in G and H is mean and standard deviation (n=3). All data except H and I are from mouse liver gene expression data set.

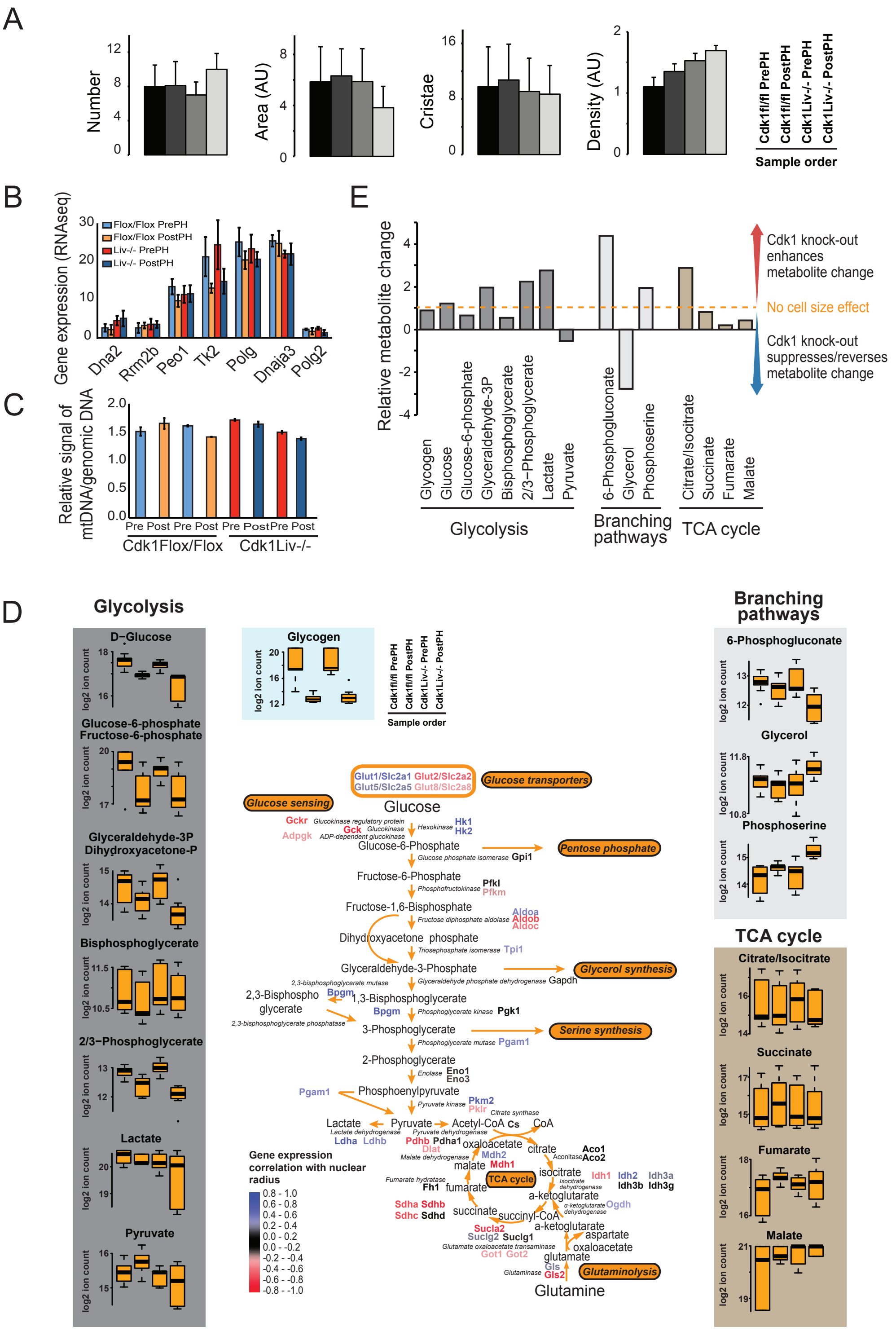

Figure S3, related to figure 3. Gene expression and metabolic changes related to glycolysis and TCA cycle. (A) Quantification of mitochondrial number per image (n=7-

10), mitochondrial area (n=47-65 mitochondria), number of mitochondrial cristae (n=44-65) and mitochondrial electron density (n=47-65). Data is mean ± SD. (B) RNAseq expression values for genes involved in mitochondrial DNA replication in the liver expression data. (C) Mitochondrial DNA to genomic DNA ratio as measured by quantitative PCR from the liver samples (n=3). (D) Detailed map of glycolysis and TCA cycle with branching biosynthesis pathways. Enzymes involved in each step are indicated next to the metabolites in the pathway map. Positive and negative gene expression correlations with cell size are in blue and red, respectively. Metabolite levels in liver samples are shown with box plots. (E) Relative metabolite changes in $Cdk^{Liv-/-}$ mice compared to $Cdk1^{Flox/Flox}$ control animals. Fold changes for postPH vs. prePH were calculated and these compared between $Cdk1^{Liv-/-}$ and control mice. Cdk1 knockout enhances metabolite changes in later stages of glycolysis with an increase in metabolite levels going to serine and glycerol synthesis. The sample order is the same in all plots (Flox/Flox prePH, Flox/Flox postPH, Liv-/- prePH and Liv-/- postPH).

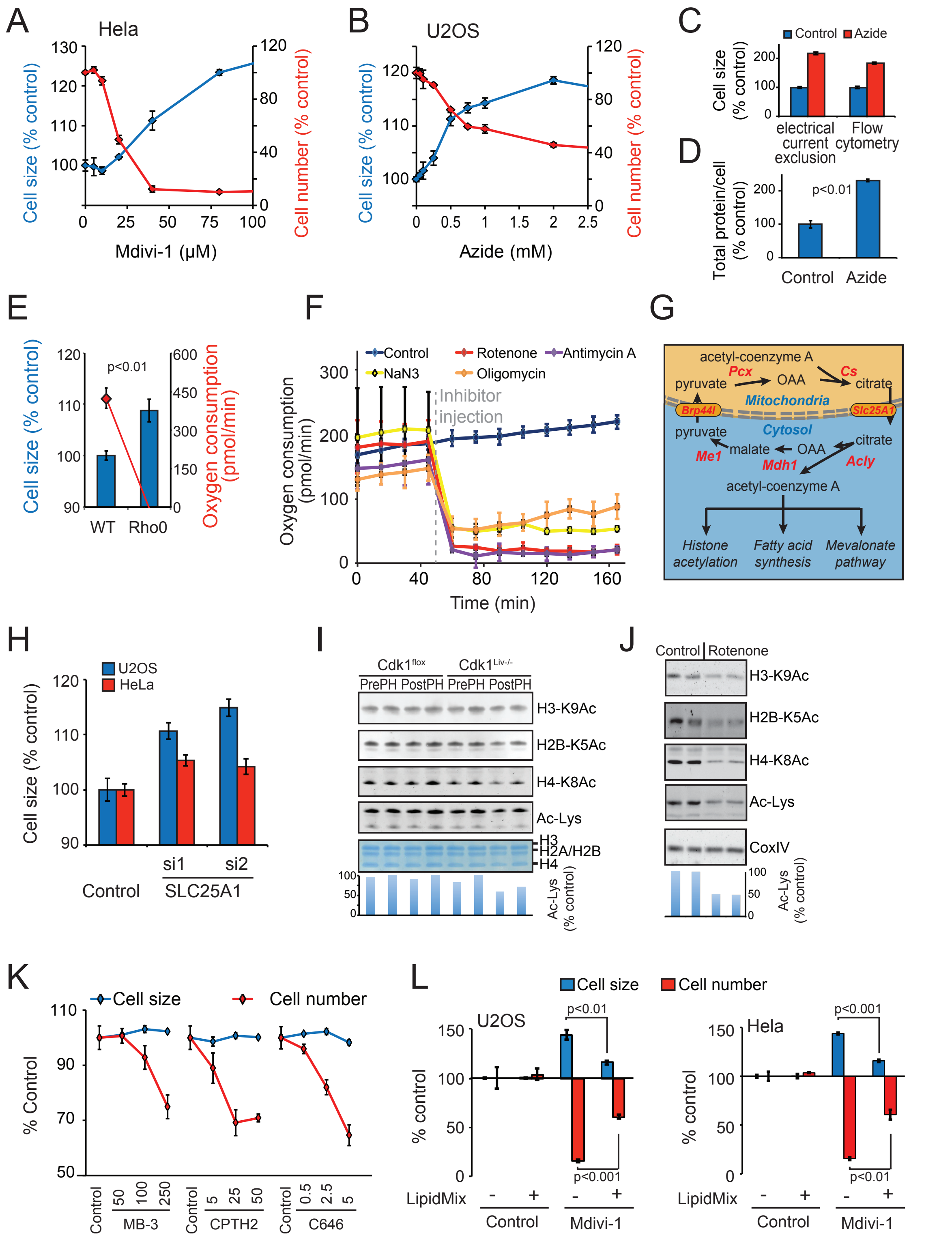

Figure S4, related to figure 4. Effects of mitochondrial targeting on proliferation and histone acetylation. (A) HeLa cell numbers (red line) and cell size (blue line) was analysed

as a function of Mdivi-1 concentration (n=3, 48h). (B) U2OS cell numbers (red line) and cell size (blue line) was analysed as a function of sodium azide concentration (n=3, 48h). (C) Comparison of cell size measurement of control and azide treated chicken DT40 cells using Casy TT (electrical current exclusion) and flow cytometry forward scatter methods. 1mM azide was used for experiment (n=3, 48h). (D) Sodium azide (1mM) increases cell size by production of more protein. U2OS were trypsinised and analysed by flow cytometry and protein concentrations were measured by Coomassie method and normalised for cell size (n=4, t-test). (E) Cell size (blue bars) and oxygen consumption (red line) of wild type (WT) and Rho0 U2OS cells (n=4, t-test). (F) Validation of OxPhos inhibitor function using oxygen consumption measurement (Seahorse assay). Inhibitors were injected at 50 minutes time point. For concentrations used, see Table S5 (n=3-5). (G) Schematic of metabolite transport between mitochondria and cytoplasm for lipid synthesis and histone acetylation. Expression of the enzymes and transporters marked with red correlate negatively with cell size. (H) Citrate transporter SLC25A1 knockdown by RNAi using two independent siRNAs in HeLa and U2OS cell (n=3, 48h). p-value is <0.01 for all SLC25A1 treatments compared to control siRNA (t-test). (I) Decrease in histone acetylation *in vivo*. Mouse liver sample histones were purified using acid extraction, run on SDS-PAGE and stained with Coomassie (lowest panel). Levels of histone acetylation were analysed by Western blotting using total acetylated lysine (Ac-Lys) or individual acetylation sites. Quantification of the total Ac-Lys signal is shown in the bottom panel. (J) U2OS cells were treated with 6 μM rotenone for 48 h. Histone acetylation was analysed by Western blot from total cellular lysates using total acetylated lysine (Ac-Lys) or individual acetylation sites. Total Ac-Lys levels were quantified and plotted (blue bars). CoxIV was used as loading control. (K) U2OS cell size (blue line) and numbers (red line) after histone acetylation inhibitor treatments. Data shown is mean with standard deviation (n=3, 28h). MB-3 is a Gcn5 inhibitor, CPTH2 inhibitor modulates the

Gcn5 network, and C646 is a competitive inhibitor of p300/CBP. (L) Rescue of Mdivi-1 induced cell size increase by 50 µl/ml LipidMix in U2OS (upper panel, 72h) and HeLa cells (lower panel, 44h) (n=3, t-test). Data shown in all panels is mean and standard deviation.

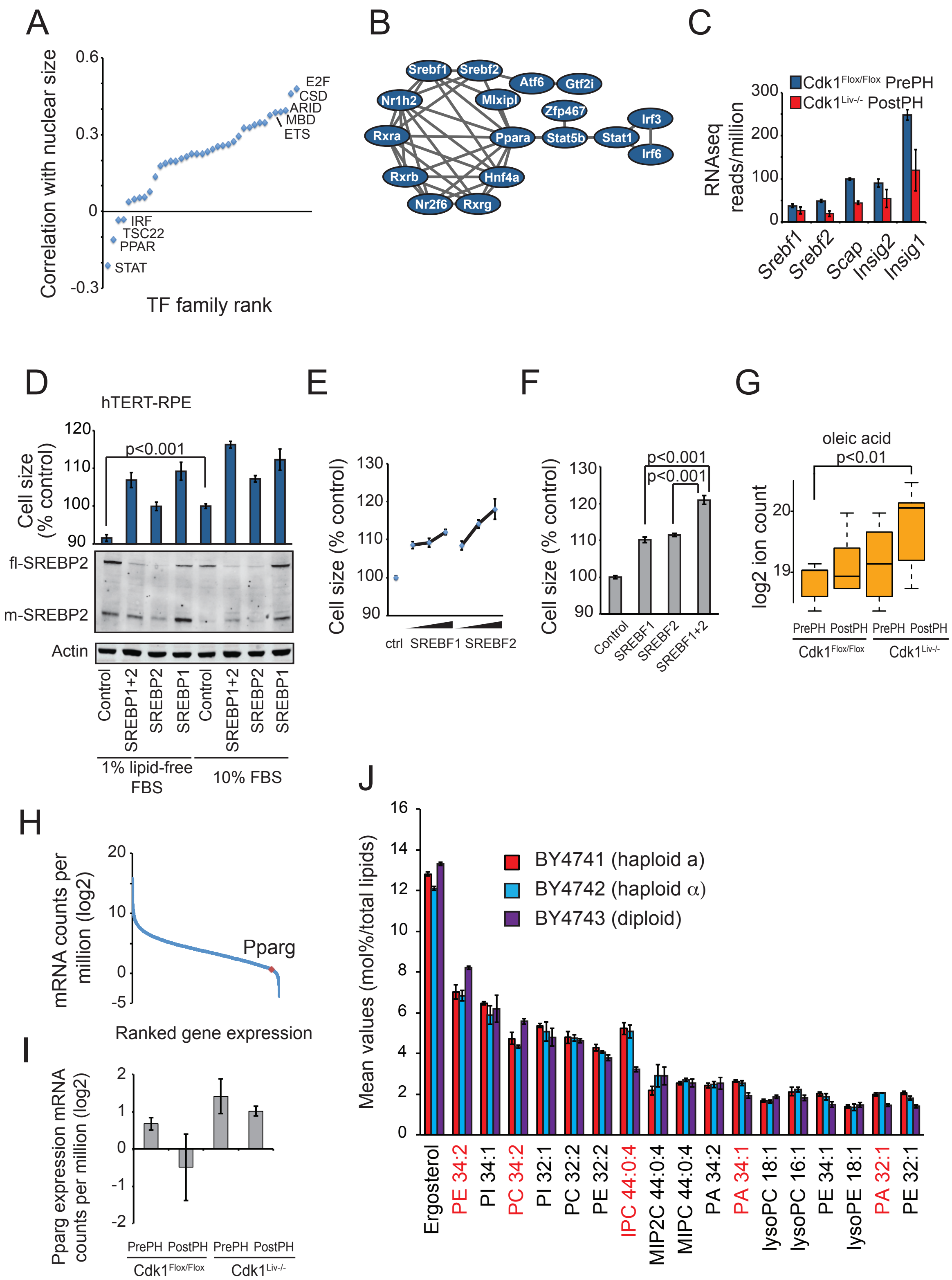
A
Correlation with nuclear size
TF family rank
E2F
CSD
ARID
MBD
ETS
IRF
TSC22
PPAR
STAT
B
Srebf1
Srebf2
Atf6
Gtf2i
Nr1h2
Mlxipl
Zfp467
Irf3
Rxra
Ppara
Stat5b
Stat1
Irf6
Rxrb
Hnf4a
Nr2f6
Rxrg
C
RNAseq reads/million
Cdk1Flox/Flox PrePH
Cdk1Liv-/- PostPH
Srebf1
Srebf2
Scap
Insig2
Insig1
D
hTERT-RPE
Cell size (% control)
p<0.001
fl-SREBP2
m-SREBP2
Actin
Control
SREBP1+2
SREBP2
SREBP1
Control
SREBP1+2
SREBP2
SREBP1
1% lipid-free FBS
10% FBS
E
Cell size (% control)
ctrl
SREBF1
SREBF2
F
Cell size (% control)
p<0.001
p<0.001
Control
SREBF1
SREBF2
SREBF1+2
G
oleic acid
p<0.01
log2 ion count
PrePH
PostPH
PrePH
PostPH
Cdk1Flox/Flox
Cdk1Liv-/-
H
mRNA counts per million (log2)
Pparg
Ranked gene expression
I
Pparg expression mRNA counts per million (log2)
PrePH
PostPH
PrePH
PostPH
Cdk1Flox/Flox
Cdk1Liv-/-
J
Mean values (mol%/total lipids)
BY4741 (haploid a)
BY4742 (haploid α)
BY4743 (diploid)
Ergosterol
PE 34:2
PI 34:1
PC 34:2
PI 32:1
PC 32:2
PE 32:2
IPC 44:0:4
MIP2C 44:0:4
MIPC 44:0:4
PA 34:2
PA 34:1
lysoPC 18:1
lysoPC 16:1
PE 34:1
lysoPE 18:1
PA 32:1
PE 32:1

Figure S5, related to figure 5. Cell size is associated with changes in lipid biosynthesis. (A) Analysis of transcription factor family expression correlation with cell size in mouse liver. Mean correlations for each family with three or more genes were calculated. (B) A network of lipogenic transcription factors correlates negatively with cell size. All transcription factors with cell size correlation <-0.3 were analysed for connectivity using STRING database. The resulting network of 17 out of these 55 transcription factors which are connected to each other is shown. (C) Expression of SREBPs and SREBP processing proteins is strongly downregulated as measured by liver RNAseq. Mean and standard deviations for the smallest and largest cells are shown. (D) Quantification of hTERT-RPE cell size changes by targeting SREBP1 and 2 in normal culture medium with 10% FBS or two days in normal medium followed by 24h in 1% delipidated FBS (n=3, 72h) to emulate the conditions used by [27]. Cell size was quantified by flow cytometry. Both full length (fl-SREBP2) and mature (m-SREBP2) forms of SREBP2 are detected by Western blotting. β-actin was used as loading control. The cell size difference between control siRNA groups under both conditions is statistically significant (p-value <0.001, t-test) as are the differences between SREBP siRNA treatments to the control siRNA (p<0.001 in all, t-test). (E) siRNA inhibition of SREBFs increases cell size in a dose dependent manner. U2OS cells were transfected with SREBF1 and SREBF2 siRNAs that were used at 6.3, 12.5, and 25 nM concentration and adjusting total siRNA concentration to 25 nM using the control siRNA (n=3, 48h). Compared to control siRNA p-value is <0.001 with all siRNA concentrations (t-test). (F) Redundant action of SREBP1 and 2 on cell size. U2OS cells were transfected with 12.5 nM of SREBP1, SREBP2 or both in combination adjusting total siRNA concentration to 25 nM using the control siRNA (n=4, 48h, t-test). (G) $Cdk1^{Liv-/-}$ mice display increase in oleate, a marker for fatty liver disease. Statistical significance was measured by ANOVA followed by Tukey's test. (H) PPARγ [Pparg], another biomarker for fatty liver, expression is not induced as

shown by RNAseq and (I) PPARγ does not increase in response to cell size changes in liver gene expression data. We conclude based on lack of PPARγ induction that the mice do not have fatty liver disease. Data shown in all panels is mean and standard deviation. (J) Comparison of lipid profiles in haploid (smaller) and diploid (larger) yeast cells. Lipidomic data is from Klose et al. [28]. Profiles for most abundant lipid species in haploid and diploid cells are plotted and names of the lipid species which appear to be differentially regulated in haploid and diploid cells are indicated with red. Two of the three most abundant phospholipids phosphatidylethanolamine (PE 34:2) and phosphatidylcholine (PC 34:2)) are increased in diploid cells versus haploid cells. Inositolphosphorylceramide (IPC44:0:4) and phosphatidic acid (PA 34:1) are less abundant in diploid cells. This data suggests that individual lipid species respond differentially to cell size changes. For naming conventions see original article [28].

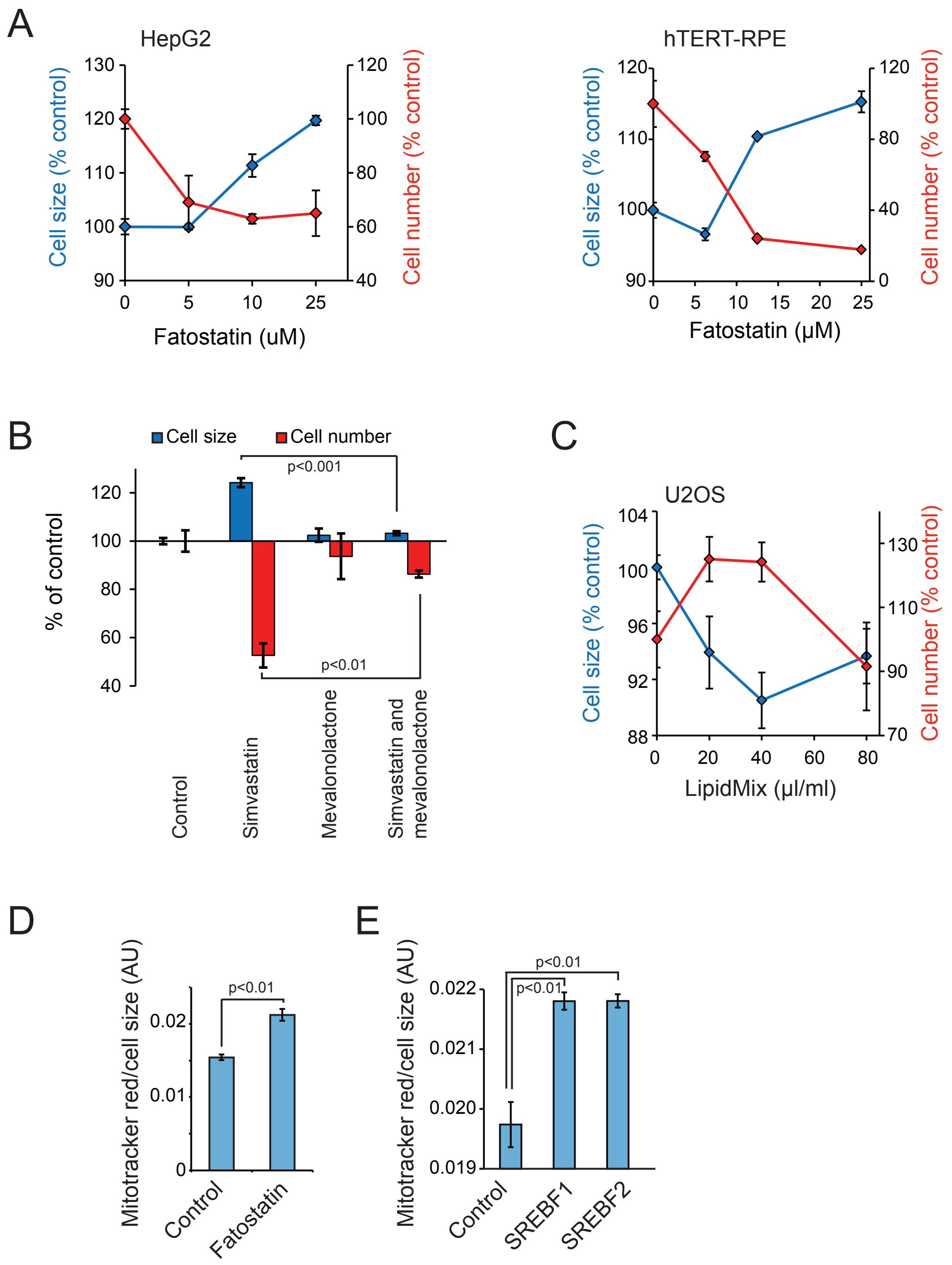

Figure S6, related to figure 6. Cell size, lipid synthesis and mitochondrial functionality are coupled. (A) Inhibition of SREBP maturation by fatostatin in HepG2 (left, 72h) and hTERT-RPE cells (right, 96h) increases cell size (n=3). (B) Rescue of simvastatin (7.5 μM) effect on cell size and cell proliferation by mevalonolactone (5 mM) in HepG2 cells (n=3, 72h, t-test). (C) Reciprocal effects of the lipid mix on U2OS cell size and cell number in

normal FBS containing medium (n=3). In normal medium, lipotoxicity is observed with high doses. (D) U2OS cells were treated with or without 20 μM fatostatin. Mitochondrial membrane potential was measured with MitoTracker Red and normalised to cell size (n=3, 54h, t-test). (E) U2OS cells were treated with control, SREBP1 or SREBP2 RNAi. Mitochondrial membrane potential was measured with MitoTracker Red and normalised to cell size (n=3, 50h, t-test). Data shown in all panels is mean ± SD.

SUPPLEMENTAL EXPERIMENTAL PROCEDURES

Mouse model

The generation of the Cdk1 conditional mice has been described previously [S1]. For the RNAseq and metabolomics analyses, $Cdk1^{Flox/Flox}$ and $Cdk1^{Liv-/-}$ mice were used before and 96 hours after partial (70%) hepatectomy. All mice used were 14 weeks old females at the time of the partial hepatectomy (PH) surgery. The liver was collected before and 96 hours after PH, snap frozen, and stored at -80°C until RNA or metabolite extraction. Nuclear size was calculated from Feulgen stained histological sections using Fiji image analysis software (version 1.46a).

RNA sequencing

Total RNA from liver samples was purified using QIAzol Lysis Reagent (Qiagen) and homogenization using Precellys homogenizer (Bertin Technologies). 15 μg total RNA was fragmented for 3 min at 70°C in RNA fragmentation buffer (Ambion). Fragmentation was terminated by cooling of the sample on ice and addition of EDTA to 17 mM final concentration. RNA sequencing was performed by tag profiling essentially as described [S2]. The resulting RNAseq library was purified using SPRI beads and sequenced at Genotypic Technology Pvt. Ltd, Bangalore, India, using 54 bp single end sequencing on Illumina GA_{IIx}.

For *Drosophila* samples, total RNA from Kc167 cells treated with dsRED control and Pop2 RNAi in triplicates for four days was purified using Qiazol and processed as above. The resulting library was sequenced at the Genepool, University of Edinburgh, using 50 bp single end sequencing on Illumina HiSeq.

The sequencing reads were mapped to transcriptome using Bowtie version 0.12.5 [S3]. For mapping, we used the longest transcript in Ensembl database. Sequencing reads matching to identical positions and having identical molecular identifier sequences were merged and the numbers of unique template molecules were counted to get expression levels [S2]. Differential gene expression was analysed using EdgeR package (mouse samples) or DEseq (*Drosophila* samples) in R.

Metabolomics analysis

For each liver, three 50 mg samples were extracted with 1.5 ml cold 50:50% methanol/H_2O using TissueLyser homogenizer (Qiagen) at 2000 rpm for 5 min. 650 µl supernatant were dried and resuspended in 1.5 ml H_2O + 0.5 ml methanol and analysed in a 1:10 dilution. For organic extraction, cell pellets from aqueous extraction were further homogenized in 1.5 ml dichloromethane/methanol and 650 µl of the resulting supernatant were dried and resuspended in 1 ml acetonitrile + 0.5 ml methanol. Samples were analysed on a 6550 Agilent QTOF mass spectrometer by untargeted flow injection analysis as described previously [S4]. Profile spectra with high mass accuracy were recorded from 50 to 1000 m/z in negative ionization mode.

For analysis, data from technical replicates (successive sample injections) was averaged. For each sample, raw intensity data was median normalised using "robust quantile normalization" function of preprocessCore package in R. This normalisation results in the relative levels of individual metabolite to the total metabolite levels of the tissue and not the

metabolite levels per cell. Ions were annotated based on accurate mass comparison against 8492 human metabolites present in the Human Metabolome Database [S5] using 1 mDa mass tolerance. The annotated metabolites were mapped on the human metabolic network derived from the KEGG database using pathway projector [S6].

Correlation of gene and metabolite expression with nuclear size

The nuclear sizes were normalized relative to those of the $Cdk1^{Flox/Flox}$ mice before hepatectomy. Pearson correlation co-efficients (r) were calculated using all samples for each gene and metabolite. Gene ontology (GO) classifications for the genes were downloaded from Ensembl database (version 62, April 2011) and used to calculate correlation histograms for each cellular subcomponent. For *Drosophila* data, GO classifications were obtained from Ensembl (version 73, September 2013).

Quantitative RT-PCR

Liver DNA was isolated and amplified in triplicates using primers TAGAGGGACAAGTGGCGTTC and CGCTGAGCCAGTCAGTGT targeting 18S rDNA sequences and mitochondrial DNA using primers ACTTCTGCCAGCCTGACCCATAGCCA and ACGCGAATGGGCCGGCTGCGTAT using Maxima SYBR Green qPCR Master Mix (ThermoFisher) with 0.1 µM ROX, 0.3 µM primers. Melting curve analyses and gel electrophoresis indicated a single PCR product. The ratio of the mitochondrial to genomic DNA values was calculated from the obtained quantification cycle (Ct) values. For RNAseq validation, total RNA used for RNAseq was reverse transcribed and amplified in triplicates with the gene-specific primer pairs (Table S6) obtained from GETPrime qPCR primer database [S7]. qPCR was performed using Maxima

SYBR Green qPCR Master Mix. Results were analysed with R (version 2.14.0) package HTqPCR (version 1.8.0) using the DCt method.

Cell culture and reagents

U2OS, HeLa, and HepG2 cells were cultured in DMEM containing 4.5g/l glucose, 10% FBS, L-glutamine and penicillin and streptomycin. hTERT-RPE cells were grown in Advanced DMEM/F-12 containing 4.5g/l glucose, 10% FBS, L-glutamine and penicillin and streptomycin and the cells were supplemented with hygromycin B (50μg/ml). DT40 cells were grown in RPMI with 10% FBS, 3% chicken serum, 1 mM β-mercaptoethanol, L-glutamine and penicillin and streptomycin.

Delipidated FBS was prepared by extracting FBS once with 2:1 vol/vol mixture of diisopropylether and n-butanol and a second extraction with diisopropylether followed by extensive dialysis against PBS using 10 000 MWCO membrane. U2OS^{rho0} cells were generated by incubating the cells with 0.1 μg/ml ethidium bromide for 3 weeks in the presence of 50 μg/ml uridine in DMEM/10% FBS. Cell size and cell number measurements were conducted using flow cytometry using Accuri C6 cytometer (Becton-Dickinson) or by electrical current exclusion method (CASY TT, Roche).

Small molecules were obtained from Sigma-Aldrich, Tocris, Santa-Cruz and Calbiochem. For small molecule data shown in Fig. 4A concentrations and solvents are shown in Table S5. For rescue experiments using LipidMixture 1 (Sigma) cells were treated with LipidMix simultaneously with chemical treatments or 24h after siRNA transfections. RNAi was performed by transfecting with 25 nM siRNA with HiPerfect (Qiagen). The siRNA sequences are shown in Table S6. Antibodies were used at their recommended concentrations and detected using infrared-dye conjugated secondary antibodies and LICOR Odyssey detection system.

For DNA content measurement, mammalian cells were stained with propidium iodide as previously described [S8]. For *Drosophila* cells, DNA staining was performed using Vybrant DyeCycle Orange live cell stain (Life Technologies) as per manufacture's instructions. For measuring mitochondrial membrane potential, MitotrackerRed (Life Technologies) was added to 150 nM final concentration for 40 min before the FACS assay and cells were washed twice with PBS before flow cytometry analysis. Oxygen consumption was measured using Seahorse XF24 instrument according to manufacturer's instructions.

Antibodies and Western Blotting

Histones were isolated using acid extraction and separated on 4-12% SDS-PAGE gels in MES buffer (LifeTechnologies). For Western blots, the following antibodies were used: Acetylated-Lysine #9441, Acetyl-Histone H3 (Lys9) (C5B11) #9649, Acetyl-Histone H2B (Lys5) #2574, Acetyl-Histone H4 (Lys8) #2594, pAMPK #2535, CoxIV #4850, GAPDH #5174, b-Actin (#4970) (all from Cell Signaling Technology). PGC-1a antibody (clone 4C1.3) was from Millipore. For analysis of OxPhos protein expression MitoProfile Total OXPHOS Rodent WB Antibody Cocktail (ab110413, Abcam) was used. Antibodies were used at their recommended concentrations and detected using infrared-dye conjugated secondary antibodies and LICOR Odyssey detection system.

Electron microscopy

Liver pieces of 1 mm^3 obtained before partial hepatectomy were immersed in 4% paraformaldehyde, 2.5% glutaraldehyde in 0.1M phosphate buffer, pH 7.4 for 5 days at 4ºC while the samples obtained after partial hepatectomy were fixed for 24 hours at 4ºC. Tissue samples were rinsed in 0.1M phosphate buffer, pH 7.4 and then 0.1M Sodium Cacodylate Trihydrate, pH 7.6 on ice. After fixation samples were exposed to Osmium fixative solution

[1% OsO_4 + 1.5% $K_3Fe(CN)_6$ in 0.1M Sodium Cacodylate Trihydrate pH 7.6] for 1 hour at RT. Samples were washed in dH_20 and dehydrated through an ascending ethanol series (from 25% to 100% ethanol) and 100 % acetone. The infiltration was continued with resin in 100% ethanol and then fresh resin overnight. Finally samples were embedded in Spurr's fresh resin and polymerized at 60ºC for 24 hours [S9]. Ultrathin sections were counterstained with 5% uranyl acetate and Reynold's lead citrate and examined with a under JEM-1010 electron microscope operated at 80 kV or JEM-2200FS at 100 kV. Images were analysed using ImageJ.

Analysis of total phospholipids

Phospholipids were measured using a colorimetric method based on the formation of a complex between phospholipids and ammonium ferrothiocyanate [S10]. Briefly, liver samples (~20mg) were homogenised in 300µl PBS and 750µl of methanol and homogenates were extracted with chloroform. Organic phase was allowed to react with $FeCl_3$-thiocyanate reagent followed by absorbance measurement at 488 nm from the organic phase. Liver samples were analysed in triplicates.

Analysis of yeast lipidomics data

Haploid and diploid lipid profiles were obtained from supplementary file from Klose *et al* [S11]**.** Individual lipid species were ranked by abundance in diploid cells and lipid levels in a and a mating pairs were compared to these. Only most abundant lipid species are shown (>1.4 mol%/total lipids). Statistical difference between yeast strains for individual lipids can not be calculated as only means and standard deviations are available in the original publication.

Statistics

The *p*-values for the obtained Pearson correlations were calculated from Student's t-distribution and were adjusted using Benjamini and Hochberg False Discovery Rate correction. Statistical significances for the differential gene expression were directly obtained using the EdgeR package (for mouse data) or DEseq package (for *Drosophila* data) in R. Cell based assay data is presented as mean ± standard deviation using two-tailed t-test. All error bars depict standard deviation. The whiskers in boxplots depict maximum and minimum values excluding outliers which are indicated with circles. The significance of differential correlation of gene expression levels with subcellular structures versus whole cell data was done using Kolmogorov-Smirnov test. Significance of the individual metabolite level changes between treatments was analysed by two-tailed t-test or ANOVA followed by Tukey's HSD test as indicated in the figure legends. The 90% confidence intervals for linear regression were calculated using *predict* function in R.

SUPPLEMENTAL REFERENCES

S1. Diril, M.K., Ratnacaram, C.K., Padmakumar, V.C., Du, T., Wasser, M., Coppola, V., Tessarollo, L., and Kaldis, P. (2012). Cyclin-dependent kinase 1 (Cdk1) is essential for cell division and suppression of DNA re-replication but not for liver regeneration. Proc Natl Acad Sci U S A *109*, 3826-3831.

S2. Kivioja, T., Vaharautio, A., Karlsson, K., Bonke, M., Enge, M., Linnarsson, S., and Taipale, J. (2012). Counting absolute numbers of molecules using unique molecular identifiers. Nature methods *9*, 72-74.

S3. Langmead, B., Trapnell, C., Pop, M., and Salzberg, S.L. (2009). Ultrafast and memory-efficient alignment of short DNA sequences to the human genome. Genome Biol *10*, R25.

S4. Fuhrer, T., Heer, D., Begemann, B., and Zamboni, N. (2011). High-throughput, accurate mass metabolome profiling of cellular extracts by flow injection-time-of-flight mass spectrometry. Anal Chem *83*, 7074-7080.

S5. Wishart, D.S., Tzur, D., Knox, C., Eisner, R., Guo, A.C., Young, N., Cheng, D., Jewell, K., Arndt, D., Sawhney, S., et al. (2007). HMDB: the Human Metabolome Database. Nucleic Acids Res *35*, D521-526.

S6. Okuda, S., Yamada, T., Hamajima, M., Itoh, M., Katayama, T., Bork, P., Goto, S., and Kanehisa, M. (2008). KEGG Atlas mapping for global analysis of metabolic pathways. Nucleic Acids Res *36*, W423-426.

S7. Gubelmann, C., Gattiker, A., Massouras, A., Hens, K., David, F., Decouttere, F., Rougemont, J., and Deplancke, B. (2011). GETPrime: a gene- or transcript-specific primer database for quantitative real-time PCR. Database : the journal of biological databases and curation *2011*, bar040.

S8. Bjorklund, M., Taipale, M., Varjosalo, M., Saharinen, J., Lahdenpera, J., and Taipale, J. (2006). Identification of pathways regulating cell size and cell-cycle progression by RNAi. Nature *439*, 1009-1013.

S9. Wisse, E., Braet, F., Duimel, H., Vreuls, C., Koek, G., Olde Damink, S.W., van den Broek, M.A., De Geest, B., Dejong, C.H., Tateno, C., et al. (2010). Fixation methods for electron microscopy of human and other liver. World journal of gastroenterology : WJG *16*, 2851-2866.

S10. Stewart, J.C. (1980). Colorimetric determination of phospholipids with ammonium ferrothiocyanate. Anal Biochem *104*, 10-14.

S11. Klose, C., Surma, M.A., Gerl, M.J., Meyenhofer, F., Shevchenko, A., and Simons, K. (2012). Flexibility of a eukaryotic lipidome--insights from yeast lipidomics. PLoS One *7*, e35063.
